# Treatment with ephrin B2 positively impacts the abnormal metabolism of human osteoarthritic chondrocytes

**DOI:** 10.1186/ar2782

**Published:** 2009-08-07

**Authors:** Steeve Kwan Tat, Jean-Pierre Pelletier, Nathalie Amiable, Christelle Boileau, Martin Lavigne, Johanne Martel-Pelletier

**Affiliations:** 1 Osteoarthritis Research Unit, University of Montreal Hospital Research Centre (CRCHUM), Notre-Dame Hospital, 1560 Sherbrooke Street East, Montreal, Quebec H2L 4M1, Canada; 2 Department of Orthopaedic Surgery, Maisonneuve-Rosemont Hospital, 5345 boulevard l'Assomption, Montreal, Quebec H1T 4B3, Canada

## Abstract

**Introduction:**

Members of the ephrin system, the ephrin receptor erythropoietin-producing hepatocellular B4 (EphB4) and its specific ligand, ephrin B2, appear to be involved in the bone remodelling process. We recently showed that their interaction inhibits the resorptive activity of human osteoarthritic (OA) subchondral bone osteoblasts. Hence, we further investigated the possible implication of these ephrin members on the catabolic/anabolic activities of human OA chondrocytes.

**Methods:**

EphB4 receptor and ephrin B2 levels were determined by quantitative PCR and immunohistochemistry, and the effects of ephrin B2 on the expression/production of factors involved in the OA process.

**Results:**

EphB4 receptors and ephrin B2 ligands are expressed and produced by human normal and OA chondrocytes. Ephrin B2 protein was found at similar levels in both cartilage types, whereas EphB4 receptor expression (*P *< 0.0001) and production (*P *< 0.01) levels were significantly increased in OA chondrocytes/cartilage. Ephrin B2 treatment significantly inhibited the interleukin (IL)-1beta, IL-6, matrix metalloproteinase-1 (MMP-1), MMP-9, MMP-13, and proteinase-activated receptor-2 (PAR-2) gene expression levels, whereas MMP-2 was unaffected, and significantly increased collagen type II, a cartilage specific macromolecule. It also inhibited the IL-1beta stimulated protein production of IL-6, MMP-1 and MMP-13.

**Conclusions:**

Our study is the first to provide data on the presence and role of ephrin B2/EphB4 receptors in human chondrocytes/cartilage. Data showed that ephrin B2 treatment positively impacts the abnormal metabolism of OA cartilage by inhibiting important catabolic factors involved in this disease at the same time as increasing anabolic activity.

## Introduction

The erythropoietin-producing hepatocellular (Eph) receptors and their ephrin ligands constitute the largest sub-family of membranous receptor tyrosine kinases. The ephrin systems are known to play crucial roles in the development of several tissues and organs, including the nervous and cardiovascular systems [1-3], and have recently been shown in bone biology. Although involved in different tissues/organs and in various phenomena, a major common role is controlling the remodelling of the extracellular matrix.

The first member of the Eph receptor family was identified and cloned in 1987 from an erythropoietin-producing hepatocellular carcinoma cell line. Eph receptors are grouped into two subclasses according to their ligand specificity. Type A receptors (EphA) generally bind preferentially to ephrins A, and type B receptors (EphB) to ephrins B. Ephrins are the ligands specific to Eph and are also divided into two subgroups that differ in their anchorage: ephrins A have a guanine nucleotide dissociation inhibitor (GDI) anchor, while ephrins B possess a single transmembrane domain.

The ephrin B ligands (ephrin B1 to B3) bind in a specific manner to their EphB receptors (Eph B1 to B6) [4-7]. Both ephrins and Eph receptors are membrane bound proteins and their interaction leads to a bidirectional Eph/ephrin signalling. Signalling through the EphB receptors is considered forward signalling and through the ephrin B ligands, reverse signalling [4-7]. The ephrin systems, and more particularly the EphB4 receptor, which has been demonstrated to bind only to its specific ligand ephrin B2 [8-11], are gaining recognition for their involvement in the control of bone homeostasis. In this tissue, osteoclasts express only ephrin B1 and B2 without any detectable EphB receptors [6], while osteoblasts express both ephrin B and EphB receptors [12]. Recently, our group [12] reported that ephrin B2 treatment could impact the abnormal metabolism of human osteoarthritic (OA) subchondral bone by inhibiting some catabolic factors contributing to its resorptive activity, thus exerting an inhibitory effect on this tissue's remodelling process. This was, to our knowledge, the first study on the possible implication of the ephrin system during the course of OA.

The present study investigating the effect of ephrin B2 in the pathogenesis of human OA chondrocytes was prompted by various findings. Firstly, since data from human OA subchondral bone [12] suggest that this ephrin system could be targeted as a specific therapeutic approach in the development of a disease modifying OA drug (DMOAD), knowing its effect on human cartilage during OA is therefore of major importance. Secondly, because subchondral bone and cartilage share a common cellular mesenchymal origin, this ephrin system may also be present and operative on chondrocytes. This could very well be considered, as the involvement of an ephrin protein in cartilage morphogenesis in chick limb bud development was previously reported [13]. Thirdly, as both bone and cartilage remodelling, although completely different processes, involve the release of catabolic factors such as matrix metalloproteases (MMPs) and pro-inflammatory cytokines, some of which are the same, investigating on human OA chondrocytes the effect of ephrin B2 on these factors is also of significance. We thus investigated the presence of ephrin B2 and its receptor EphB4 on human OA chondrocytes as well as the functional consequences of ephrin B2 treatment on these cells on both catabolic and anabolic mediators. Very interestingly, data showed that chondrocyte treatment by ephrin B2 positively impacts human OA chondrocyte metabolism.

## Materials and methods

### Specimen selection

Normal human cartilage was obtained from individuals within 12 hours of death (mean age ± SD, 50 ± 16), and OA specimens (69 ± 8) from patients undergoing total knee arthroplasty. All patients were evaluated as having OA according to American College of Rheumatology clinical criteria [14]. At the time of surgery the patients had symptomatic disease requiring medical treatment in the form of analgesics, non-steroidal anti-inflammatory drugs (NSAIDs), or selective cyclooxygenase (COX)-2 inhibitors. None had received intra-articular steroid injections within three months prior to surgery. The institutional Ethics Committee Board of the University of Montreal Hospital Centre approved the use of the human articular tissues.

### Chondrocyte culture

Chondrocytes were released from full-thickness strips of cartilage followed by sequential enzymatic digestion at 37°C, as previously described [15]. Cells were seeded at high density (10^5 ^cells/cm^2^) and cultured to confluence in Dulbecco's modified Eagle's medium (DMEM) (Wisent Inc., Saint-Bruno, QC, Canada) supplemented with 10% heat-inactivated fetal calf serum (FCS; PAA Laboratories Inc., Etobicoke, ON, Canada) and an antibiotics mixture (100 units/ml of penicillin base and 100 μg/ml of streptomycin base) (Wisent Inc.) at 37°C in a humidified atmosphere. To ensure phenotype, only first-passage cultured chondrocytes were used.

The effects of factors were assessed on OA chondrocytes by pre-incubating cells in DMEM/0.5% FCS (Gibco-BRL) for 24 hours followed by 18 hours (for mRNA determination) and 72 hours (for protein determination) incubation with fresh culture medium containing the factors under study. The incubation periods for gene expression level and protein production were determined following preliminary experiments, which demonstrated maximum effects at 18 hours for gene expression and 72 hours for protein production. The effect of ephrin B2 on OA chondrocytes was assessed by incubating the cells with either 50 or 100 ng/ml of human recombinant ephrin B2 (Abnova, Taipei, Taiwan) in the absence (gene expression) or presence (protein production) of interleukin (IL)-1β (100 pg/ml; Genzyme, Cambridge, MA, USA). The concentrations of the ephrin B2 ligand were chosen according to the literature including a previous publication from our group on another human cell type [12]. Moreover, these concentrations were further verified by performing a preliminary experiment on human chondrocytes using increasing concentrations of ephrin B2: 10, 50, 100 and 200 ng/ml. Data showed that 50 and 100 ng/ml give the maximal effect.

### RNA extraction, reverse transcription (RT), and real-time polymerase chain reaction (PCR)

Total cellular RNA from human chondrocytes was extracted with the TRIzol™ reagent (Invitrogen Corporation, Burlington, ON, Canada) according to the manufacturer's specifications. The RNA was quantitated using the RiboGreen RNA quantitation kit (Invitrogen Corporation, Carlsbed, CA, USA). The RT reactions were primed with random hexamers as previously described [16]. The primer sequences were as shown in Table 1.

**Table 1:** Primer Sequence

Gene	Sense	Antisense
EphB4 receptor	5'-CACAGTCATCCAGCTCGTG	5'-ATCGGATGGGAATCTTTCC
ephrin B2	5'-TTCGACAACAAGTCCCTTTG	5'-CGAGTGCTTCCTGTGTCTC
IL-1β	5'-TTAGGAAGACACAAATTGC	5'-TGGGCAGACTCAAATTCCAG
IL-6	5'-CACCTCTTCAGAACGAATTG	5'-CTAGGTATACCTCAAACTCC
PAR-2	5'-GAAGCCTTATTGGTAAGGTTG	5'-CAGAGAGGAGGTCAGCCAAG
MMP-1	5'-CTGAAAGTGACTGGGAAACC	5'-AGAGTTGTCCCGATGATCTC
MMP-2	5'-CACTGTTGGTGGGAACTCAG	5'-GTGTAAATGGGTGCCATCAG
MMP-9	5'-CCTTCACTTTCCTGGGTAAG	5'-CCATTCACGTCGTCCTTATG
MMP-13	5'-CTTAGAGGTGACTGGCAAAC	5'-GCCCATCAAATGGGTAGAAG
Collagen type II	5'AGTTTCAGGTCTCTGCAGGT	5'-CCAGAAGCACCTTGGTCTC
GAPDH	5'-CAGAACATCATCCCTGCCTCT	5'-GCTTGACAAAGTGGTCGTTGAG

Real-time quantitation of mRNA was performed as previously described [16] in the Rotor-Gene RG-3000A (Qiagen, Valencia, CA, USA) with the 2× Quantitect SYBR Green PCR Master Mix (Qiagen) according to the manufacturer's specifications. The data were given as a threshold cycle (C_T_) and calculated as the ratio of the number of molecules of the target gene/number of molecules of GAPDH. The primer efficiencies for the test genes were the same as for the GAPDH gene.

### Immunohistochemistry

Cartilage specimens were processed for immunohistochemical analysis. Slides were prepared as previously described [17] and further incubated with a blocking serum (Vectastain ABC assay; Vector Laboratories Inc., Burlingame, CA, USA) for 60 minutes, after which they were blotted and then overlaid with the primary antibody of goat anti-human EphB4 receptor (15 μg/ml; R&D Systems, Minneapolis, MN, USA) or rabbit anti-human ephrin B2 ligand (5 μg/ml; Sigma-Aldrich, Oakville, ON, Canada) for 18 hours at 4°C. Slides were incubated with the second antibody (anti-goat or anti-rabbit IgG; Vector Laboratories) for one hour at room temperature, followed by staining with the avidin-biotin-peroxidase complex method (Vectastain ABC assay; Vector Laboratories, Inc.). The colour was developed with 3,3'-diaminobenzidine (DAKO Diagnostics Inc., Mississauga, ON, Canada) containing hydrogen peroxide. Slides were counterstained with eosin. Sections were examined under a light microscope (Leitz Orthoplan; Leica Inc., St. Laurent, QC, Canada).

Three control procedures were performed: (i) omission of the primary antibody, (ii) substitution of the primary antibody with an autologous preimmune serum, and (iii) absorption with the human recombinant EphB4 receptor (R&D Systems) or ephrin B2 at 20× and 50× respectively. Controls showed only background staining.

**Figure 1:**
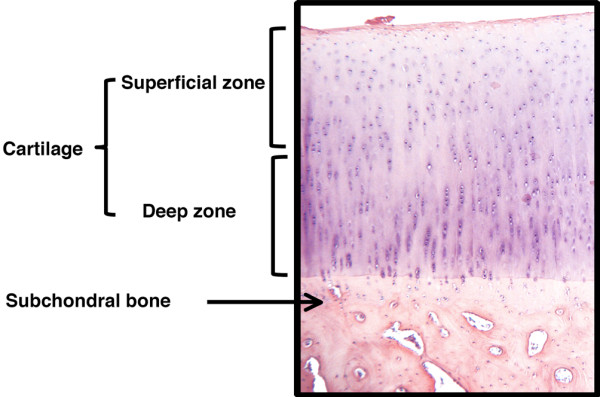
Human cartilage subdivided into two zones: superficial zone (superficial and upper intermediate layers) and deep zone (lower intermediate and deep layers). The subchondral bone plate is also represented.

Positive cells were quantified as previously described [17]. In brief, three sections of each specimen were examined (40×; Leitz Orthoplan) from either the superficial zone of the cartilage (the superficial and upper intermediate layers) or the deep zone (the lower intermediate and deep layers) as illustrated in Figure 1, scored, and the resulting data integrated as a mean for each specimen. The final results were expressed as the percentage of chondrocytes staining positive for the antigen (cell score) with the maximum score being 100%. Each slide was subjected to evaluation by two observers with >95% degree of agreement.

### Determination of interleukin and MMP production

IL-1β, IL-6, MMP-1, and MMP-13 were determined by specific ELISAs (R&D Systems) in the culture media. All determinations were performed in duplicate for each cell culture.

### Statistical analysis

Data are expressed as the mean ± SEM of independent specimens. Statistical significance was assessed by the 2-tailed Student's t-test, and *P* values ≤ 0.05 were considered significant.

## Results

### Ephrin B2 and EphB4 receptor expression and production

**Figure 2:**
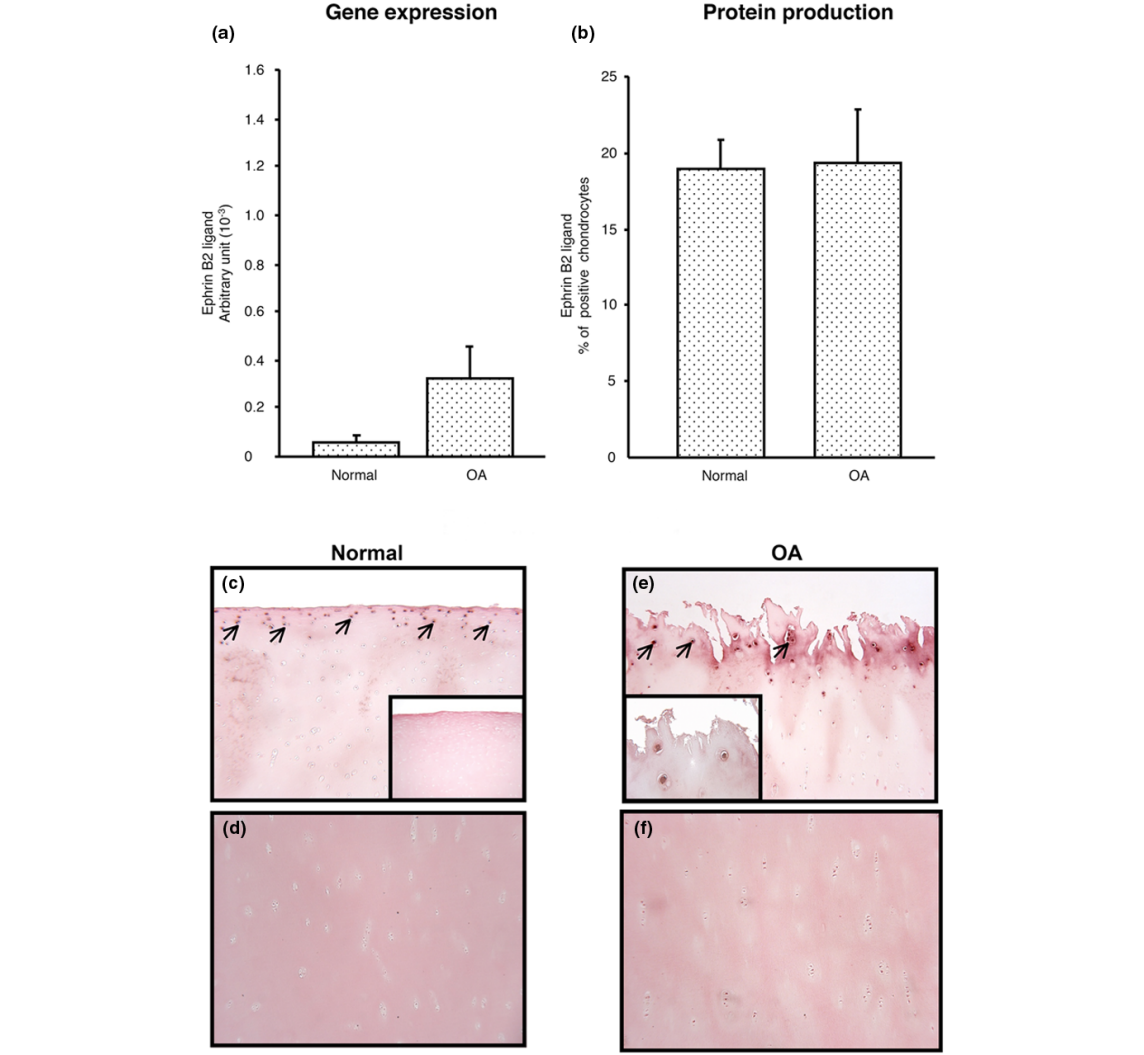
Ephrin B2 **(a)** gene expression level in human normal (n = 5) and osteoarthritic (OA) (n = 4) chondrocytes, and **(b)** protein production as analyzed following immunohistochemistry as described in Materials and Methods in the superficial zone in normal (n = 4) and OA (n = 6) cartilage. Of note, the arbitrary unit of the ephrin B2 ligand gene is expressed as × 10^-3^. **(c) **Representative immunohistological sections showing ephrin B2 in the superficial zone of human normal and **(d) **OA cartilage and **(e) **the deep zone of human normal and **(f) **OA cartilage. The insets represent in **(c) **a negative control done with immunoabsorption with only background staining and in **(e) **a higher magnification of positive cells stained for ephrin B2. **c**, **d**, **e**, **f **and inset in **c**: original magnification ×100, and inset in **e**: original magnification ×400. Arrows indicate stained chondrocytes. Statistical significance assessed by Student's t-test revealed no difference.

Data showed that the ephrin B2 expression level was slightly higher in OA chondrocytes (n = 4) compared to normal (n = 5) (Figure 2a). However, the protein production of ephrin B2 was similar in normal (n = 4) and OA chondrocytes (n = 6) (Figures 2b–f). In both cartilage types, ephrin B2 was localized in the superficial zone (Figures 2c, 2d) and no positive cells were detected in the deep zone (Figures 2e, 2f). The Figure 2c inset represents a negative control done with immunoabsorption of ephrin B2 showing only background staining, and Figure 2e inset a higher magnification of some positive cells stained for ephrin B2.

**Figure 3:**
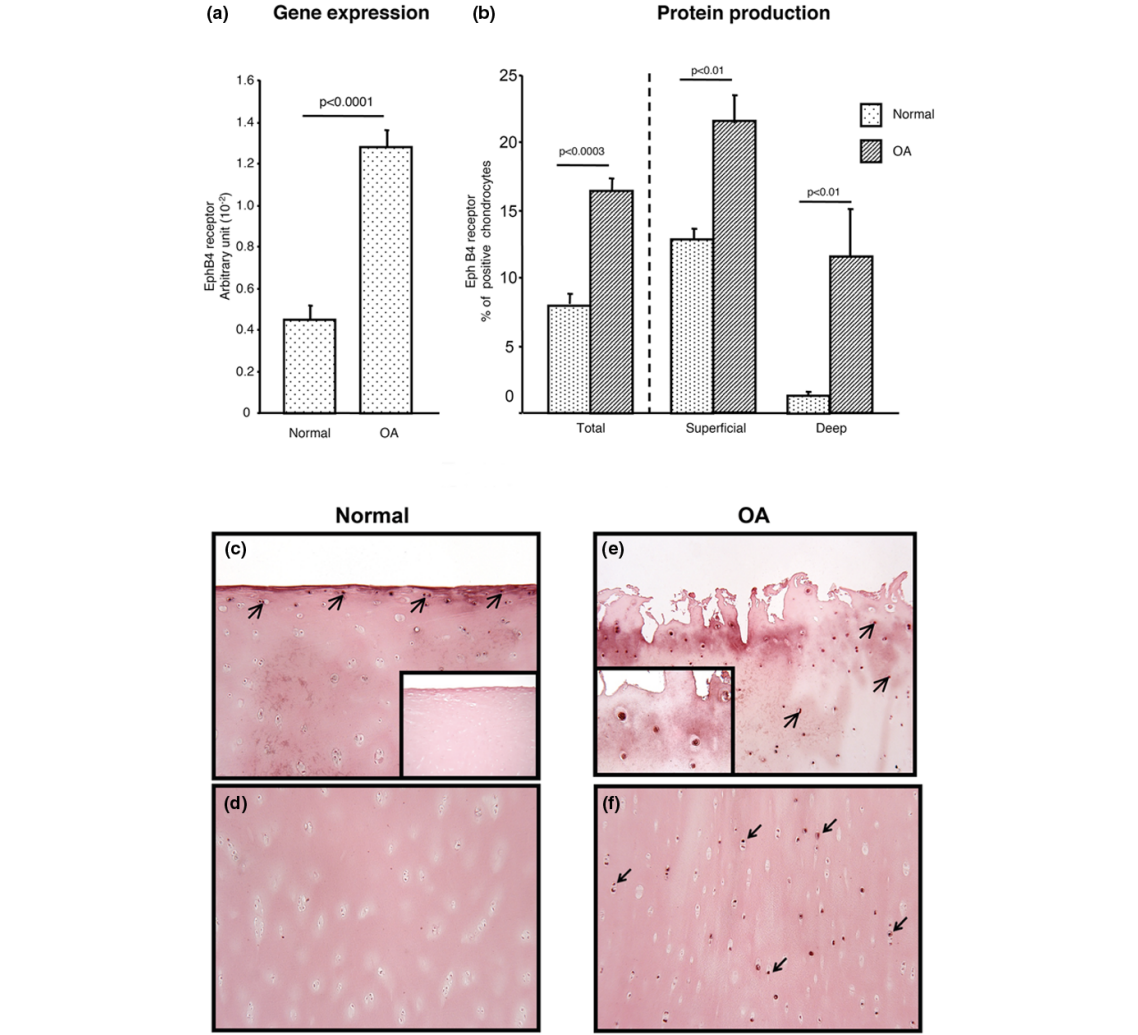
EphB4 receptor **(a)** gene expression level in human normal (n = 5) and osteoarthritic (OA) (n = 8) chondrocytes, and **(b)** total protein production as analyzed following immunohistochemistry as described in Materials and Methods in normal (n = 4) and OA (n = 4) cartilage or in the superficial or deep zones of the cartilage. Of note, the arbitrary unit of the EphB4 receptor gene is expressed as × 10^-2^. **(c) **Representative immunohistological sections showing EphB4 receptor in the superficial and **(d) **deep zone of human normal cartilage and in the **(e) **superficial and **(f) **deep zone of OA cartilage. The insets represent in **(c) **a negative control done with immunoabsorption with only background staining and in **(e) **a higher magnification of positive cells stained for ephrin B2. **c**, **d**, **e**, **f **and inset in **c**: original magnification ×100, and inset in **e**: original magnification ×400. Arrows indicate stained chondrocytes. Statistical significance was assessed by Student's t-test and *P* values are as underlined.

In contrast to ephrin B2, the gene expression level of the EphB4 receptor was significantly elevated (*P *< 0.0001) in OA chondrocytes (n = 8) compared to normal (n = 5) (Figure 3a). EphB4 receptor protein production was also found at a significantly higher level (*P *< 0.0003) in OA (n = 4) than in normal (n = 4) cartilage (Figure 3b). In normal cartilage, the EphB4 receptor was produced only in the superficial zone (Figures 3c, 3d), whereas in OA, EphB4 receptor positive chondrocytes were found throughout the cartilage (Figures 3e, 3f), with a statistically significant increase (*P *< 0.01) found in both zones (Figure 3b). As for the ephrin B2 above, the inset in Figure 3c represents a negative control done with immunoabsorption of EphB4 receptor showing only background staining, and the Figure 3e inset a higher magnification of positive cells stained with the EphB4 receptor antibody.

### Functional consequences of ephrin B2 treatment

**Figure 4:**
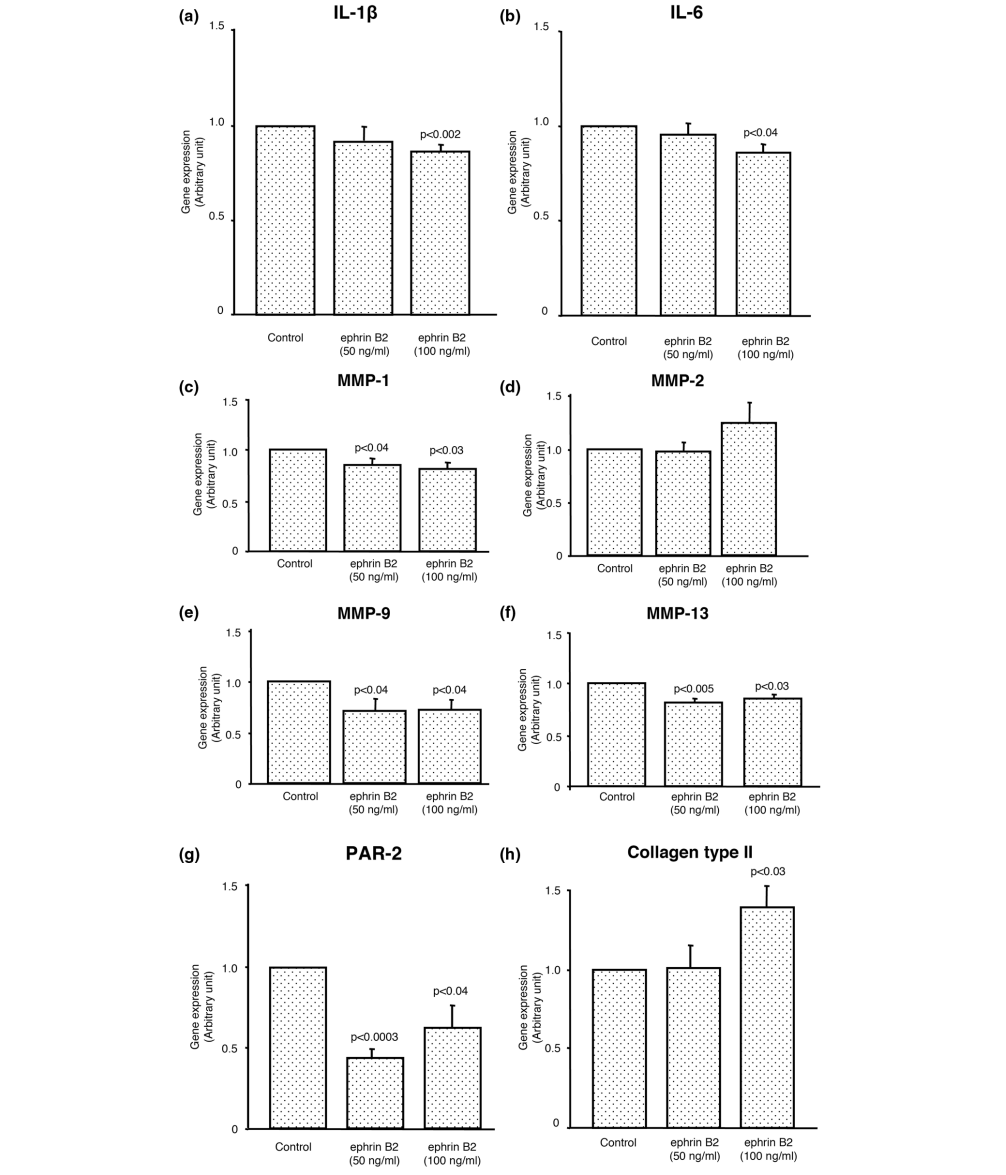
Effect of ephrin B2 activation of the EphB4 receptor on human osteoarthritic chondrocytes (n = 8) on the gene expression level of **(a)** IL-1β, **(b)** IL-6, **(c)** MMP-1, **(d)** MMP-2, **(e)** MMP-9, **(f)** MMP-13, **(g)** PAR-2, and **(h)** collagen type II. Cells were incubated for 18 hours. Data are expressed as the mean ± SEM of arbitrary unit over the control which was attributed a value of 1. Statistical significance was assessed by Student's t-test and *P* values are versus control.

We then investigated the OA chondrocytes (n = 8) upon treatment with ephrin B2 (50 and 100 ng/ml), the modulation of some catabolic and anabolic factors known to be involved in the physiological/pathophysiological chondrocyte processes. These were IL-1β, IL-6, MMP-1, MMP-2, MMP-9, and MMP-13, the proteinase-activated receptor-2 (PAR-2), a receptor involved in inflammatory pathways and recently shown to play an important role in OA [17,18], and the collagen type II. Data revealed that ephrin B2 treatment led to a pattern of reduced expression of several catabolic factors. Both pro-inflammatory cytokines, IL-1β and IL-6, were significantly inhibited (*P *< 0.002, *P *< 0.04 respectively) (Figures 4a, 4b); the reduction was dose dependent and significance reached at 100 ng/ml of ephrin B2. MMP-1, MMP-13, and MMP-9, but not MMP-2, were also significantly decreased with ephrin B2 at both concentrations (50, 100 ng/ml) tested (Figures 4c, 4d, 4e, 4f). A similar significant inhibitory effect was obtained for PAR-2 expression upon treatment with the ephrin B2 ligand at 50 and 100 ng/ml (Figure 4g). Interestingly, ephrin B2 at 100 ng/ml significantly increased (*P *< 0.03) the expression level of collagen type II (Figure 4h).

In addition, experiments were done with OA chondrocytes (n = 6 to 8) incubated in the absence or presence of ephrin B2 at 50 and 100 ng/ml with or without IL-1β at 100 pg/ml and the protein production of IL-6, MMP-1 and MMP-13 determined. Data first showed that ephrin B2 alone had no effect on the basal levels of IL-6, MMP-1 or MMP-13 (data not shown), possibly due to the fact that the production of these factors by the OA chondrocytes was at the limit of detection. The basal level of IL-1β was also very low, yet slightly higher than the limit of detection with a mean value of 14.6 ± 4.4 ng/mg protein recorded. The treatment with ephrin B2 at 100 ng/ml abolished such detection, indicating that ephrin B2 decreases the protein synthesis of this cytokine.

Since *in vivo *OA pathophysiology is characterized by the presence of IL-1β, protein production of these factors was further determined in the presence of this cytokine. As MMP-2 and MMP-9 are not truly modulated by IL-1β [19,20], they were not studied. Data as represented in Table 2 showed that the significant stimulatory effect of IL-1β on the production of IL-6, MMP-1, and MMP-13 was inhibited by ephrin B2, with a statistically significant effect obtained for IL-6 (*P *< 0.05) and MMP-13 (*P *= 0.05) at 100 ng/ml ephrin B2.

**Table 2:** Protein production of IL-6, MMP-1 and MMP-13 after a 72 hour incubation period on human osteoarthritic chondrocytes

	IL-6 (μg/mg protein)	MMP-1 (μg/mg protein)	MMP-13 (μg/mg protein)
Control	0.3 ± 0.1	12.7 ± 3.1	0.5 ± 0.1
IL-1β (100 pg/ml)	3.6 ± 0.6 *(*P *< 0.002)	62.7 ± 17.7 *(*P *< 0.02)	3.5 ± 1.0 *(*P *< 0.01)
IL-1β+ephrin B2 (50 ng/ml)	2.2 ± 0.4	41.2 ± 5.7	1.9 ± 0.3
IL-1β+ephrin B2 (100 ng/ml)	2.1 ± 0.4 †(*P *< 0.05)	38.6 ± 5.5	2.0 ± 0.3 †(*P *= 0.05)

Data are expressed as mean ± SEM.

* Indicates statistically significant difference compared to control values, and † compared to IL-1β values.

## Discussion

Osteoarthritis is a debilitating disease resulting from a complex degradative mechanism in the articular joint. Although considerable advancement has been made towards a better understanding of the pathophysiological pathways that occur during the OA process, much remains to be accomplished in the development of an effective DMOAD that would reduce or stop the disease progression. In this context, identifying new candidates able to target several joint tissues (cartilage, subchondral bone and synovial membrane) is extremely attractive.

Our group recently showed, in human OA subchondral bone osteoblasts [12], that ephrin B2 treatment induces a reduction in the abnormal remodelling process as well as in several catabolic factors involved in bone matrix alterations. These data suggest that ephrin B2 could exert a protective effect on structural changes in OA articular tissues, which makes this ephrin system an attractive and interesting therapeutic target in OA. Since the cartilage also demonstrates a remodelling of its extracellular matrix during the disease process and the ephrin system is known to control extracellular matrix, we explored the implication of this ephrin system in human OA cartilage metabolism and identified factors targeted in the diseased tissue. We investigated the presence of ephrin B2 and the EphB4 receptor in human articular cartilage/chondrocytes and the effects of treatment with ephrin B2 on human OA chondrocytes. This is the first time that this system has been studied in chondrocytes, and our data revealed important new information about its mechanisms of action in cartilage.

The first finding was that the EphB4 receptor is differentially expressed and produced by normal and OA chondrocytes/cartilage, with a significantly increased expression level in OA compared to normal. In contrast to normal, OA cartilage showed not only a significantly increased number of chondrocytes in the superficial zone producing the EphB4 receptor, but its production was also extended to the deep zone. Ephrin B2, however, did not appear to be modulated in human OA cartilage. The data showing a higher level of EphB4 receptors in OA chondrocytes combined with those showing ephrin B2 treatment decreased inflammatory/catabolic factors and increased collagen type II suggest that exogenous ephrin B2 treatment could be of interest in limiting the degradation involved in abnormal cartilage breakdown.

Data first showed that ephrin B2 treatment significantly decreased the expression levels of the proinflammatory cytokines IL-1β and IL-6 which are highly involved in the severity and perpetuation of this disease [21-25]. Experiments also demonstrated a similar inhibition of the collagenases MMP-1 and MMP-13, which are closely linked to the degradative properties in cartilage because of their activity not only on collagen but also on a wide range of non-collagenous extracellular macromolecules [26-34]. Data also showed that IL-1β protein production as well as the IL-1β-induced synthesis of IL-6, MMP-1 and MMP-13 by OA chondrocytes was markedly reduced by ephrin B2, thus strengthening the hypothesis suggesting its *in vivo *beneficial and protective effect.

Although MMP-1 and MMP-13 are the most important members of this family in relation to cartilage degradation, some other MMPs including the gelatinases have also been suggested to be involved in the OA pathological process [19,20,35-38]. We therefore investigated the effect of the activation of this ephrin system on MMP-2 and MMP-9. Data revealed a significant inhibition of MMP-9, but not of MMP-2. The lack of effect on MMP-2 is not surprising and is consistent with the literature indicating the greater significance of MMP-9 in joint diseases than MMP-2. Indeed, knockout mouse experiments revealed that the absence of MMP-9, but not of MMP-2, reduces arthritis progression [39]. Positive correlation between the production of MMP-9, but not of MMP-2, was also found with rapid destruction in human hip OA [40,41]. Moreover, the plasma level of MMP-9, but not of MMP-2, is upregulated in OA compared to normal [42]. The differences between these two MMPs could be due to the differential pathways in cell signalling. Indeed, such differences were seen, although on other articular cell types, in synovial and meniscal tissues in which the production of latent and active forms of MMP-9 was mediated partly through Jun N-terminal kinase (JNK) and p38, whereas MMP-2 was not modulated by such pathways. Moreover, experiments carried out on monocytes and macrophages derived from rheumatoid arthritis demonstrated that the role of CD147 in MMP production and cell invasion enhanced MMP-9 production through extracellular signal-related kinase 1/2 (Erk1/2) and JNK, whereas MMP-2 production was not modulated at all. Altogether, these data strengthen our current observation about the differential modulation of MMP-2 and MMP-9 by this ephrin system [43,44].

In the joint, the inflammatory response is a major component in sustaining the progression of OA [45]. In that respect, a factor belonging to the PARs, PAR-2, has been shown to be involved in arthritic inflammatory pathways, and data generated by using a PAR-2 gene knockout mouse in the adjuvant-induced arthritis model demonstrated its important role in chronic arthritis [46-48]. It was also suggested that PAR-2 could be an upstream regulator of pro-inflammatory cytokines in articular tissue cells and responsible for their upregulation [49]. Moreover, PAR-2 was recently found to be closely linked to cartilage remodelling in human OA [17,18]. Interestingly, this study showed that treatment with ephrin B2 inhibits this pro-inflammatory factor.

Finally, in order to complement the effect of this ephrin system in OA chondrocytes, we also investigated whether ephrin B2 exerts an effect on a cartilage specific macromolecule, collagen type II. Data indeed showed this system's ability to induce collagen type II expression by human OA chondrocytes. Altogether, these experiments demonstrated that ephrin B2 treatment on human OA chondrocytes leads to decreased catabolic/inflammatory properties at the same time as having an anabolic effect.

As well described in the literature, the ephrin B2 ligand and EphB4 receptors are present at the cell membrane and Hattori et al [50] recently proposed that membranous ephrin ligands could be cleaved by some proteases. It would therefore be very appealing to further explore such shedding mechanism and identify the protease(s) responsible for the cleavage in articular tissues. Such cleavage would increase the level of soluble ephrin B2 in OA extracellular matrix, enabling it to better exert its effect on its specific receptor, thus contributing to a protective effect on cartilage matrix.

Hence, in human OA cartilage treatment with ephrin B2 could act at two different levels: (i) by limiting the extent of matrix degradation through the inhibition of the most important interleukin and MMP involved in OA cartilage breakdown, as well as PAR-2, another inflammatory factor, and the IL-1β-induced catabolic factors, and (ii) by promoting the production of the cartilage specific macromolecule collagen type II. Thus, data from this study on human chondrocytes and the previous one on subchondral bone [12] strongly suggest this ephrin system as a potential and very attractive therapeutic target for OA.

## Conclusions

In conclusion, the data showing that treatment of OA chondrocytes by ephrin B2 down-regulates various catabolic factors in cartilage at the same time as increasing a major anabolic factor, collagen type II, are of significance. These data indicate that treatment of OA patients with ephrin B2 or that an increase in this endogenous ligand could be an interesting approach in the development of a specific therapeutic agent able to act on more than one tissue of the joint.

## Abbreviations

COX: cyclooxygenase; C_T_: threshold cycle; DMEM: Dulbecco's modified Eagle's medium; DMOAD: disease modifying osteoarthritis drug; Eph: erythropoietin-producing hepatocellular; EphB4: ephrin receptor erythropoietin-producing hepatocellular B4; Erk1/2: extracellular signal-related kinase 1/2; FCS: fetal calf serum; GDI: guanine nucleotide dissociation inhibitor; IL: interleukin; JNK: Jun N-terminal kinase; NF-κB: nuclear factor kappa B; NSAID: non-steroidal anti-inflammatory drug; OA: osteoarthritis; PAR-2: proteinase-activated receptor-2.

## Competing interests

The authors declare that they have no competing interests.

## Authors' contributions

SKT helped to design the study, acquire data, analyse and interpret data, prepare the manuscript and participated in the statistical analysis. JMP and JPP helped to design the study, and prepare the manuscript. NA helped to acquire data and analyse and interpret data. CB helped to analyse and interpret data and participated in the statistical analysis. ML helped to acquire data. All authors read and approved the final manuscript.
